# Incorporating Deep Learning With Word Embedding to Identify Plant Ubiquitylation Sites

**DOI:** 10.3389/fcell.2020.572195

**Published:** 2020-09-30

**Authors:** Hongfei Wang, Zhuo Wang, Zhongyan Li, Tzong-Yi Lee

**Affiliations:** ^1^Warshel Institute for Computational Biology, The Chinese University of Hong Kong, Shenzhen, China; ^2^School of Life Sciences, University of Science and Technology of China, Hefei, China; ^3^School of Life and Health Sciences, The Chinese University of Hong Kong, Shenzhen, China

**Keywords:** ubiquitylation, plant, word embedding, deep learning, transfer learning, convolutional neural network

## Abstract

Protein ubiquitylation is an important posttranslational modification (PTM), which is involved in diverse biological processes and plays an essential role in the regulation of physiological mechanisms and diseases. The Protein Lysine Modifications Database (PLMD) has accumulated abundant ubiquitylated proteins with their substrate sites for more than 20 kinds of species. Numerous works have consequently developed a variety of ubiquitylation site prediction tools across all species, mainly relying on the predefined sequence features and machine learning algorithms. However, the difference in ubiquitylated patterns between these species stays unclear. In this work, the sequence-based characterization of ubiquitylated substrate sites has revealed remarkable differences among plants, animals, and fungi. Then an improved word-embedding scheme based on the transfer learning strategy was incorporated with the multilayer convolutional neural network (CNN) for identifying protein ubiquitylation sites. For the prediction of plant ubiquitylation sites, the proposed deep learning scheme could outperform the machine learning-based methods, with the accuracy of 75.6%, precision of 73.3%, recall of 76.7%, F-score of 0.7493, and 0.82 AUC on the independent testing set. Although the ubiquitylated specificity of substrate sites is complicated, this work has demonstrated that the application of the word-embedding method can enable the extraction of informative features and help the identification of ubiquitylated sites. To accelerate the investigation of protein ubiquitylation, the data sets and source code used in this study are freely available at https://github.com/wang-hong-fei/DL-plant-ubsites-prediction.

## Introduction

As one of the most important posttranslational modification (PTM) processes, ubiquitylation is a modification process in which one or more ubiquitin molecules covalently bind to substrate proteins under the action of a series of enzymes (E1, E2, E3) (Weissman, 2001). The ubiquitin–proteasome pathway (UPP) is the most important protein degradation pathway in eukaryotic cells and participates in various physiological processes, including transcription regulation, cell cycle, apoptosis, DNA damage repair, metabolism, and immunity (Tu et al., 2012). Moreover, its abnormal regulation is often accompanied with the occurrence of diseases such as cancer, neurodegenerative diseases, and liver diseases (Hoeller et al., 2006; Popovic et al., 2014; Yamada et al., 2018). UPP is closely related to plant physiology, and many studies have proved that ubiquitin–proteasome degradation is involved in plant growth and development, abiotic stress, plant metabolism, and biological stress (Lu et al., 2011;Marino and Rivas, 2012).

Because of the functional significance of ubiquitylation, the identification of new ubiquitylation sites in proteins is highly significant. However, wet laboratory experimental validations are often time consuming and expensive (Nguyen et al., 2016). In contrast, computation-based identification methods, which combine big data and advanced algorithms, can provide an alternative strategy for ubiquitylation site prediction with fast speed and low cost. The population of high-throughput proteomics experiment technology promotes large-scale identification of ubiquitin-conjugated peptides and, then, provides a very large dataset for automatic recognition of ubiquitination sites (Nguyen et al., 2015). Recently, numerous machine learning methods have been proposed for automatic prediction of ubiquitination sites. The Ubipred (Tung and Ho, 2008) is the first online tool that employed the physical and chemical properties of amino acids surrounding ubiquitination sites as features and integrated with support vector machine (SVM) to predict the ubiquitination sites. Then other machine learning methods, such as the k-nearest neighbor and random forest, are also used for ubiquitination site prediction (Radivojac et al., 2010; Cai et al., 2012; Xiang et al., 2013; Jyun-Rong et al., 2016). The hCKSAAP-UbSite (Chen et al., 2013) employed the idea of the composition of k-spaced amino acid pair (CKSAAP), which considers amino acid pair composition features of a specific position. Qiu et al. (2015) believing, through the simple observation of the composition of amino acids, that the sequence order of proteins may be ignored, utilized the pseudo-amino acid composition (PseAAC) to reserve these essential features and developed the iUbiq-Lys. The ubiquitylated protein data are collected from various eukaryotic species, and, considering the features of species evolution, Ubisite (Huang et al., 2016) proposed the position-specific scoring matrices (PSSM), which are calculated through PSI-BLAST. As a promising structural data modeling approach, the deep learning method can extract features from original data automatically without feature engineering, thus some potential and essential features will not be ignored. He et al. (2018) employed the deep learning approach on ubiquitination site prediction and received a well performance on their testing set.

However, the pattern differences between the ubiquitylated proteins of these species are not clear. To the best of our knowledge, no related work focuses on ubiquitylation prediction model development for a particular species. In this work, we first analyzed the pattern differences of ubiquitylated proteins between plants, animals, and fungus. Then an improved word-embedding training scheme based on transfer learning was proposed, connecting with the multilayer convolutional neural network (CNN) for plant ubiquitylation site prediction.

## Materials and Methods

**FIGURE 1:**
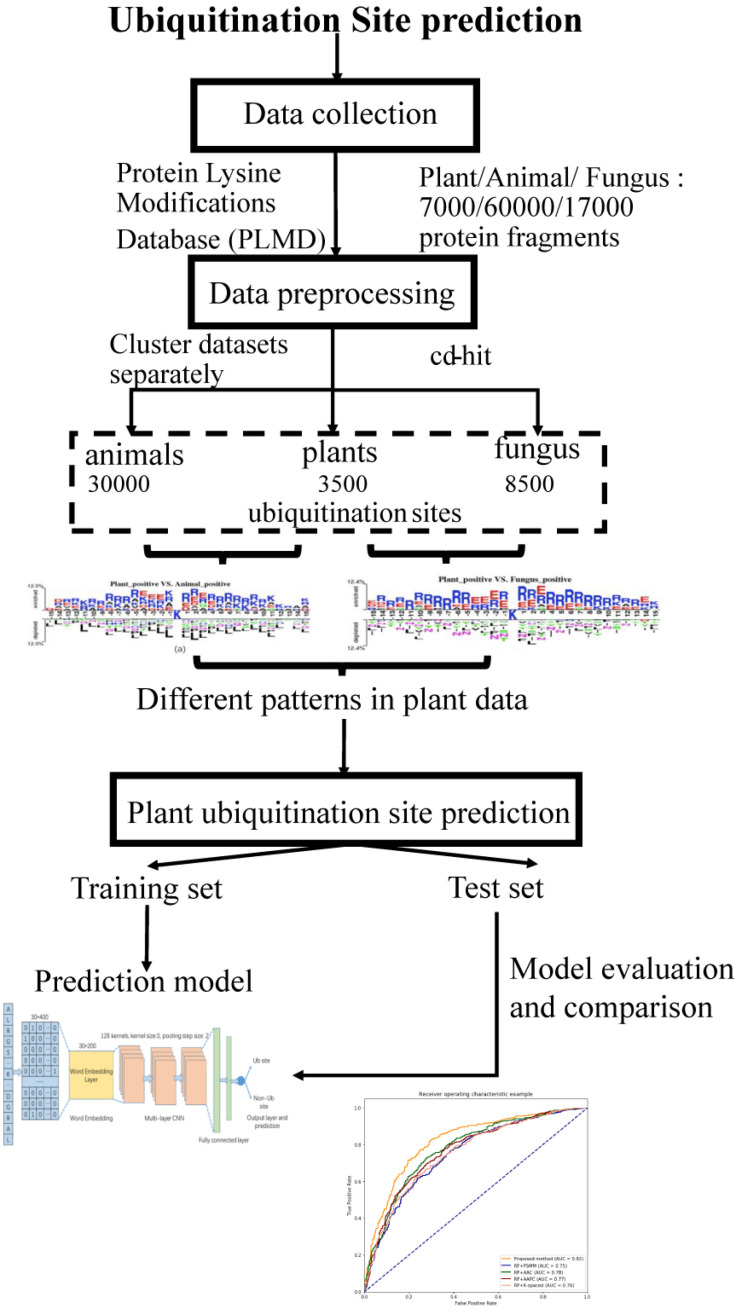
Schematic diagram of the workflow for this study.

The workflow of this study is described in Figure 1. We collected ubiquitination sites data from the Protein Lysine Modifications Database (PLMD) (Xu et al., 2017), which includes data collected from plants, animals, and fungus. In order to understand the pattern differences of ubiquitylated protein sequences between these species, feature investigations of three species were conducted. Several important sequence features were compared and analyzed to illustrate the pattern differences between plants and other species. Then a novel transfer learning-based word-embedding training scheme was proposed in which two steps of training were conducted. The original plant protein sequence was used for pretraining of the word2vec network through the skip-gram model, with the optimized parameter transfer as the initial weights of embedding layer and fine-tuning with the subsequent layers together. The trained word-embedding layer captured the sequence features of the plant protein and was appropriated to ubiquitination site prediction at the same time. The multilayer CNN was employed as a classifier and achieved acceptable performance for plant ubiquitination site prediction. Sufficient experiments illustrated that the proposed method outperforms the conventional method on both cross-validation and the independent testing set.

### Data Collection and Preprocessing

In this study, the ubiquitination protein sequence is collected from the PLMD database (Xu et al., 2017); the original data contains 121,742 ubiquitination sites from 25,103 proteins. We selected ubiquitination sites from *Arabidopsis thaliana*, *Oryza sativa* subsp *indica*, and *O. sativa* subsp *japonica* for the plant subset, ubiquitination sites from *Homo sapiens* and *Mus musculus* for the animal subset, and *Saccharomyces cerevisiae* for the fungus subset. To construct positive data of modeling, a sliding window with a length of 31 was used to intercept the protein sequences with ubiquitylated lysine residues in the center, where 31 equals 15 amino acids from each side of the lysine residue plus one lysine residue. If the upstream or downstream residues of a protein are less than 15, the lacking residue is filled with a pseudoresidue X. Then, the sequence fragments that contained a window length of 31 amino acids were centered at the lysine residue without annotation of the ubiquitination and were regarded as the negative data of modeling (non-ubiquitylated lysines). We removed the redundant protein fragments to eliminate homology bias using the CD-HIT (Li and Godzik, 2006) with 30% identity to ensure that none of the segments had a larger than 30% pairwise identity in both positive and negative peptides. There are too many negative peptides compared to the positive peptides. In order to keep the data balanced, we selected the same number of negative peptides randomly as positive peptides. Finally, we obtained 7,000 protein fragments for the plant subset, 60,000 protein fragments for the animal subset, and 17,000 protein fragments for the fungus subset.

We obtained 3,500 ubiquitination sites from plants after the preprocessing steps through CD-HIT tools, and then, we selected 3,500 negative samples randomly to keep the data balanced. In this work, we employed both the independent testing and cross-validation method to evaluate the performance of the proposed model. We selected 1,500 protein fragments randomly from the 7,000 samples as the independent testing set, which were used to evaluate the tuned model. In addition, we utilized the 10-fold cross-validation method to test the model performance using the remaining 5,500 samples. The original dataset was randomly partitioned into 10 equal-sized subsamples; a single subsample was retained as the validation data for testing the model, and the remaining nine subsamples were used as training data. The cross-validation process was then repeated 10 times, with each of the 10 subsamples used exactly once as the validation data.

### Feature Investigation

#### Amino Acid Composition

As an important sequence feature, amino acid composition (AAC) can reflect which kind of amino acid is more likely to appear around the ubiquitylated lysine. In this work, we calculated the AAC feature of each peptide using the following equation:

$$A_{r}=\frac{N_{r}}{N}r=1,2,3,\ldots ,20$$

where *N*_r_ denotes the number of amino acid *r*, and *N* denotes the length of the protein fragments.

#### Amino Acid Pairwise Composition

In order to understand the efforts of amino acid complexes for ubiquitination in these species, we calculated the relative frequencies of all possible dipeptides in the sequence. The elements of the feature vector are defined as:

$$D_{r,s}=\frac{N_{r,s}}{N},r,s=1,2,\ldots ,20$$

where *N*_r,s_ denotes the count of the dipeptide *r*,*s*, and *N* represents the total number of dipeptides in the encoded segment. Consequently, a 400-dimensional vector would be obtained for each segment. Then, heat maps were used to illustrate the dipeptide composition difference between the positive and negative samples, and the value of each pixel was calculated using the following equation:

$$P_{r,s}=\ln\frac{\sum D_{\text{positive}}}{\sum D_{\text{negative}}}$$

#### Positional Weighted Matrix

Then, we made the positional weighted matrix (PWM) to illustrate the pattern differences of the amino acid distribution around the ubiquitylated lysine between the positive and negative samples, and three heat maps were plotted for these three species, respectively. We define a two-dimensional matrix for each fragment as *M*^*i*^, whose horizontal axis denotes the positions of protein fragments, and the central position is the targeted lysine, while the vertical axis denotes all these 20 kinds of amino acids. The final PWM for comparison of the positive and negative samples is calculated through the following equation:

$$M_{\mathrm{PA}}=\ln\frac{\sum M_{\text{positive}}^{i}}{\sum M_{\text{negative}}^{i}}$$

#### Two Sample Logo

We also employed the Two Sample Logo (Vacic et al., 2006) web server to calculate and visualize the differences between ubiquitylated fragments from different species. Two Sample Logos can be used to determine statistically significant residues around various active sites, protein modification sites, or to find differences between two groups of sequences that share the same sequence motif.

### Sequence Encoding

Compared with the traditional machine learning and statistical computation method, the deep learning approach can extract features automatically from original data without feature engineering (Schmidhuber, 2015). Thus, transferring the amino acid sequences to quantification vectors, which can be processed by a computer program directly, is important (Hua and Quan, 2016). Word embedding is a set of techniques in natural language processing in which words from a vocabulary are represented as vectors using a large corpus of text as the input.

Generally, there are two main word-embedding techniques used in sentence processing. The first method is embedding layer in neural network (Neishi et al., 2017); the essence of embedding layer is a fully connected neural network, which can map the one-hot sequence to a dimensionally specified vector. Some popular deep learning frameworks have predefined functions for this layer. The process of parameter learning of this method is supervised; the parameters are updated with subsequent layers during the learning process under the supervision of a class label. Several PTM site prediction works are based on this scheme. Another word-embedding technique is Word2vec (Mikolov et al., 2013), where similar vector representations are assigned to the words that appear in similar contexts based on word proximity as gathered from a large corpus of documents. After training on a large corpus of text, the vectors representing many words show interesting and useful contextual properties. The training of word2vec is unsupervised because the class label does not participate in the learning process.

In this work, inspired by pretraining and fine-tuning mechanisms of transfer learning, we first employed the original plant protein sequences as training data and pretrained the embedding layer based on the unsupervised skip-gram algorithm. The optimized embedding layer can map each amino acid from a sequence into a vector. The Euclidean distance of vectors can reflect the relative position information of an amino acid. So, the embedding layer can capture the spatial features of the amino acids in the pretraining process. Then, the optimized parameters are transferred as the initial weights of the embedding layer, and fine tuning is done with the subsequent layers together under the supervision of the label of fragments. By contrast, the traditional word-embedding methods often initialize weights randomly and are trained together subsequently, which may ignore the sequence position information. Compared with traditional word-embedding methods, the proposed scheme is more appropriate for plant ubiquitination site prediction.

**FIGURE 2:**
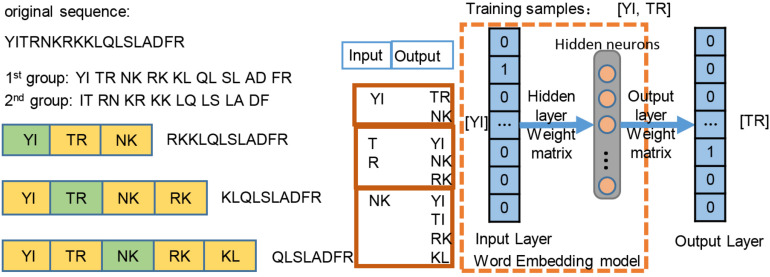
Word2vec training process of the bigram pattern.

We employed the skip-gram method (Du et al., 2018) on the construction of word2vec mapping network. The protein sequences were represented as a collection of counts of n-grams, in which *n* adjacent amino acids were recognized as a word. Inspired by the idea of Hamid and Friedberg (2018), the length of the gram of 1, 2, 3 was tested in our work, and *n* = 2 was optimal, leading to 20^2^ = 400 bigrams. Figure 2 simply shows the representation learning for bigrams with the skip-gram training. For each protein sequence, we created two sequences by starting the sequence from the first and second amino acids, so that we can consider all of the overlapping bigrams for a protein sequence. We generated the training instances using a context window of size ±2, where we took the central word as input and used the surrounding words within the context window as outputs. The neural network architecture for training was used on all of instances, then a 200-dimensional vector for each bigram was generated by the neural network. The trained hidden layer weights were transferred as the initial parameters of the embedding layer in the proposed ubiquitination site prediction model.

### Word2vec With Convolutional Neural Network

**FIGURE 3:**
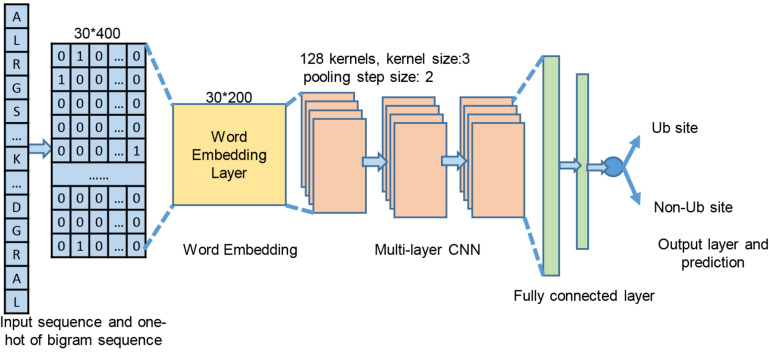
Proposed deep structure for ubiquitination site prediction model.

After sequence encoding, one-dimensional CNN was employed to take the bigram encoding vectors as input and predict the label of this fragment whose lysine in the central position can be ubiquitylated or not. The forward calculation of the CNN deep structure is an automatic feature extraction and selection process in each layer. As shown in Figure 3, each bigram maps into a 20-dimensional vector so that a sequence of 31 amino acid residues is represented as a 30 × 20 matrix, which was denoted as X. The next step is the convolutional layer where the filters were used to extract sequence features from the encoding matrix. The process is denoted as

$$C_{1}=\delta_{1}\left(W_{1}\times X+b_{1}\right)$$

where δ_1_ is the rectified linear (Relu) function, *W*_1_ denotes the weights of the convolution kernel, and *b*_1_ is the bias of this layer. Then, the max pooling function is used for downsampling procedure to reduce the feature dimension.

$$C_{1,\text{out}}=\max\mathrm{pooling}\left(C_{1}\right)$$

The CNN deep structure contained three same sequentially connected blocks, and each block covered a convolution layer with the Relu as its activation function and a max pooling layer. The number of convolution kernels was set as 128, and the convolution kernel size was set at 3. The size of the max pooling windows was 2. Two fully connected layers with 128 and 64 neurons, respectively, are used to integrate features. The output layer contained a single neuron and ends with sigmoid activation to calculate the output x of this layer as

$$\text{Sigmoid}\left(x\right)=\frac{1}{1+e^{-x}}$$

The backward process of the CNN network is backward propagation, which updates and gets optimal parameters with the following binary cross-entropy loss function.

$$\text{BCE}\left(\hat{y},y\right)=-\frac{1}{N}\sum_{i=1}^{n}\left[y_{i}\cdot \log\left(\hat{y_{i}}\right)+\left(1-y_{i}\right)\cdot \log\left(1-\hat{y_{i}}\right)\right]$$

During the training of the CNN models, the dropout units (the drop rate was set at 0.5) were added after each max pooling layer in the convolutional layer, which are usually required for generalization on unseen data and to avoid overfitting.

### Implementation and Training Parameters

The proposed model was achieved through the Keras framework under the Python language. We set the initial learning rate as 0.001, and the RMS prop optimization method was used with β = 0.9. We initialized the weights of the convolutional network randomly with a Gaussian distribution (μ = 0, σ = 0.01). The batch size is 500, and 120 epochs were conducted for each training. All the experiments were performed on a server equipped with Geforce RTX 2080 Ti.

## Results

### Comparison of Features Between Species

#### Amino Acid Composition

**FIGURE 4:**
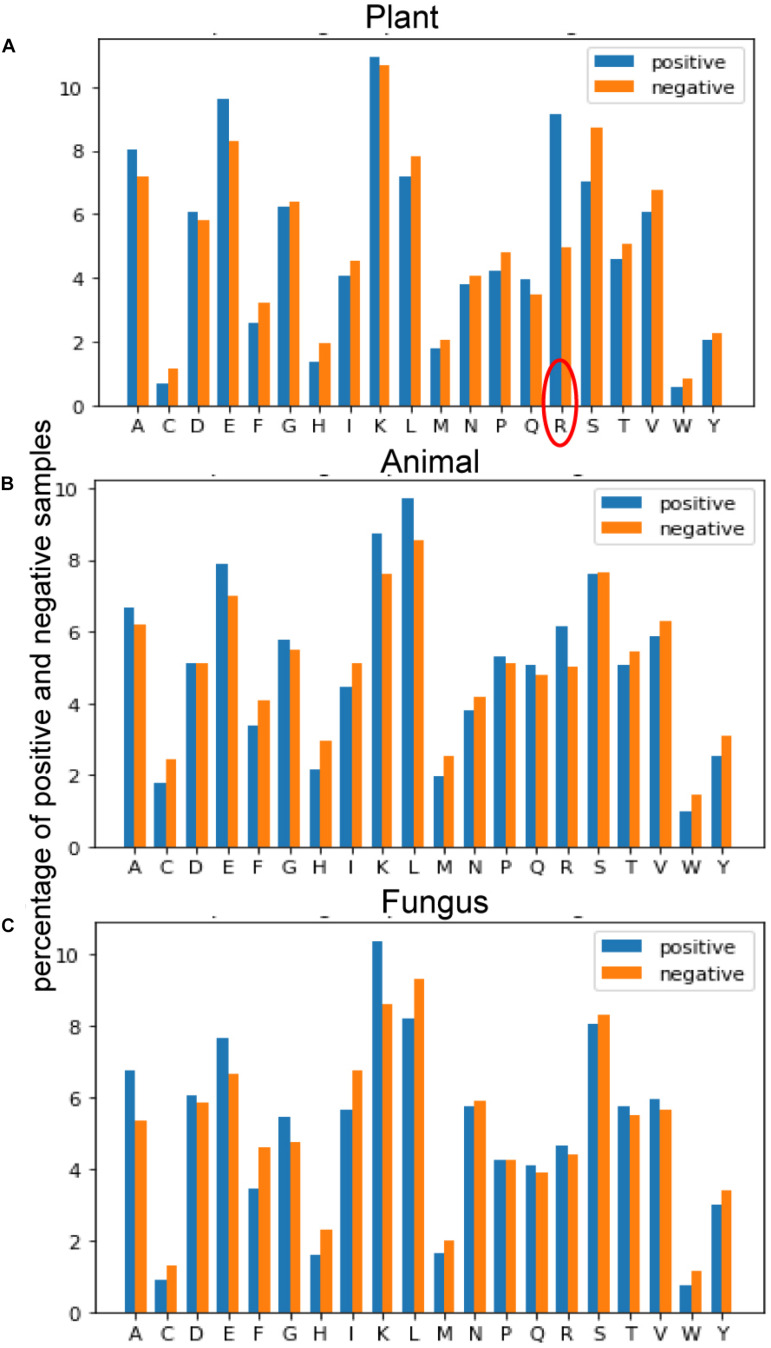
Comparison of the amino acid composition (AAC) features between three species. **(A)** Comparison of AAC features between positive and negative samples of plants. **(B)** Comparison of AAC features between positive and negative samples of plants. **(C)** Comparison of AAC features between positive and negative samples of plants.

Figure 4 provides the average of positive and negative segments, respectively, and a histogram for each species was plotted. We can analyze the amino acid composition differences between the positive and negative segments to show different patterns of these spices. For the plant subset, the average percentage of arginine (R) in ubiquitylated protein fragments is doubled in non-ubiquitylated protein segments. By contrast, the arginine differences between ubiquitylated and non-ubiquitylated segments in animals and fungi are not obvious, although the figure of positive samples is 0.7% higher than the negative samples in animals. For animals and fungi, the average percentage of lysine (K) in the positive protein segments is about 1% higher than in the negative samples, and this difference is not obvious in plant samples. What is more, the percentage of leucine (L) in ubiquitylated proteins of animal is 1% higher than in non-ubiquitylated samples; this finding is contrary in plants and fungi. So, the amino acid composition shows really different patterns in different species.

#### Amino Acid Pairwise Composition

**FIGURE 5:**
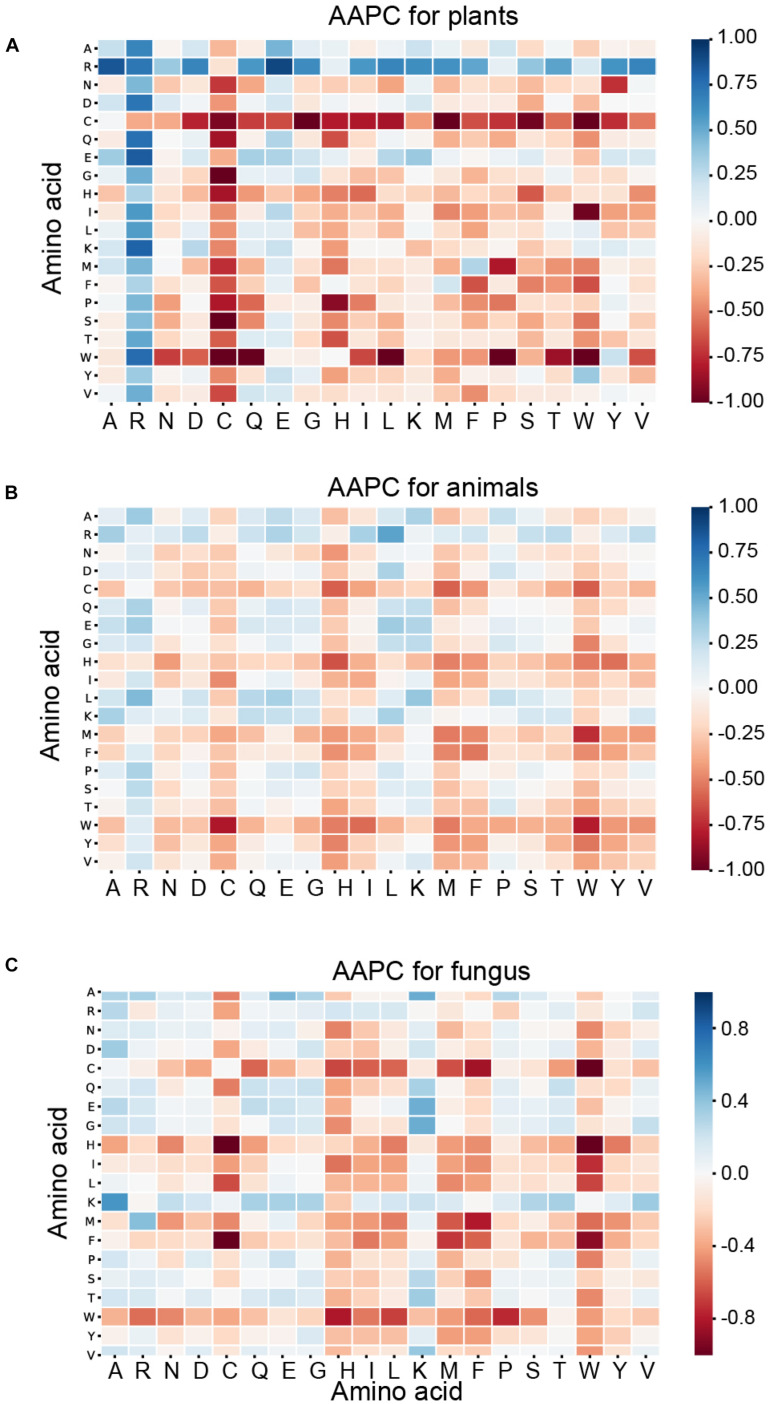
Heatmaps for the amino acid pairwise composition (AAPC) features of three species. **(A)** Heatmap for the AAPC features of plants. **(B)** Heatmap for the AAPC features of animals. **(C)** Heatmap for the AAPC features of fungi.

As shown in Figure 5, the blue pixels mean this dipeptide is more likely to appear around ubiquitylated lysine than in non-ubiquitylated lysine, while the red means it is less likely to appear in ubiquitylated fragments than in the negative samples. The darker the color, the greater the difference. For the ubiquitination of plants, cysteine (C) is less often composed with other amino acids, such as glycine (G), methionine (M), serine (S), and tryptophan (W). The *P*_r,s_ of these dipeptides are less than −0.75, which denotes that the distribution of these dipeptides presents obvious differences between the ubiquitylated and non-ubiquitylated fragments in the plant subset. In addition, the pairs that contain arginine (R), especially with alanine (A) and glutamic acid (E), are more likely to appear around the ubiquitylated lysine with *P*_r,s_ of more than 0.5. However, these phenomena above do not appear in the animal and fungus subsets. For the animal subset, the value of *P*_r,s_ for the majority of the amino acid combinations range from −0.25 to 0.25, which means that there are no obvious differences between the positive and negative samples, expect that tryptophan (W), combined with cysteine (C), and methionine (M), is less likely to appear in ubiquitylated peptides than in non-ubiquitylated samples. For the fungus subset, cysteine (C), combined with methionine (M), and histidine (H), as well as tryptophan (W), combined with cysteine (C), histidine (H), and phenylalanine (F), are less likely to appear in ubiquitylated peptides than in non-ubiquitylated samples. The *P*_r,s_ of these amino acid combinations are less than −0.7. The statistical differences of the AAPC feature between the ubiquitylated and non-ubiquitylated fragments show very different patterns in three different species.

#### Positional Weighted Matrix

**FIGURE 6:**
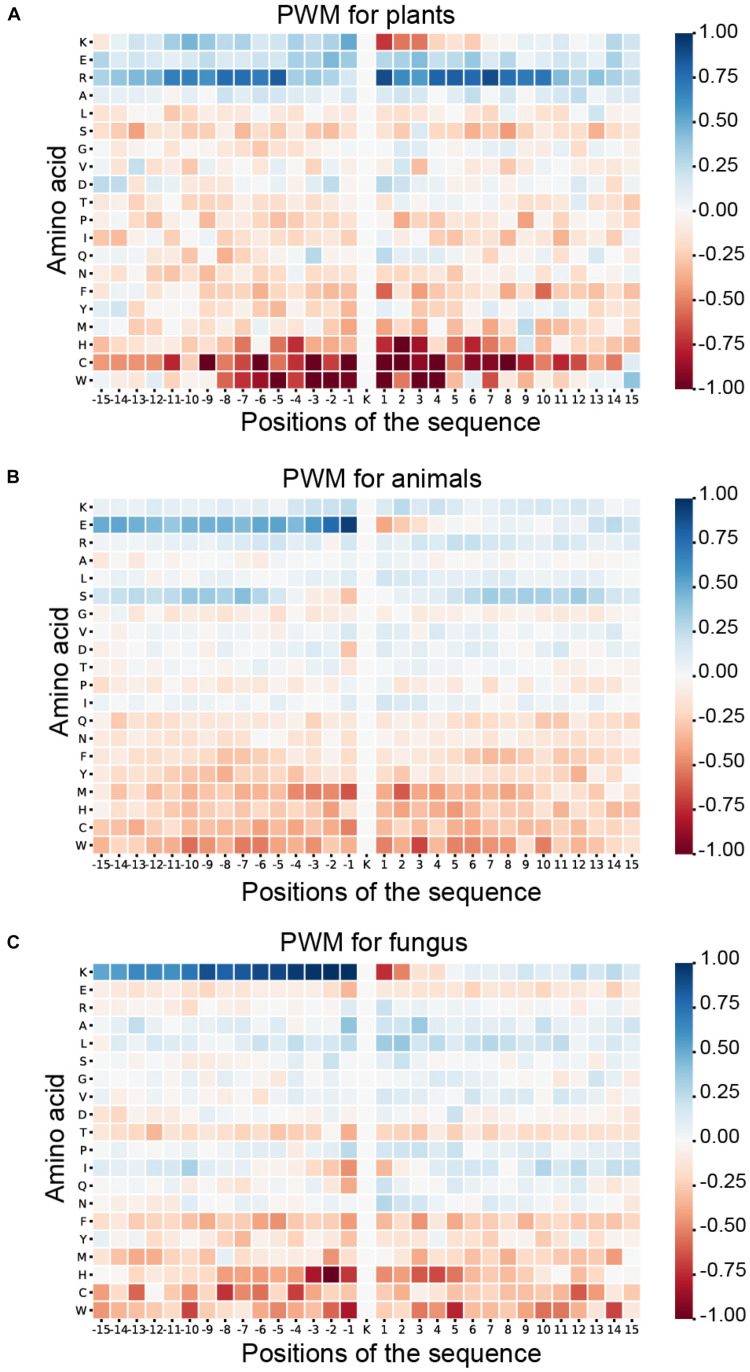
Heatmaps for the positional weighted matrix (PWM) features of three species. **(A)** Heatmaps for the PWM features of plants. **(B)** Heatmaps for the PWM features of animals. **(C)** Heatmaps for the PWM features of fungi.

As shown in Figure 6, blue means that the amino acid is more likely to appear in this position of ubiquitylated fragments, and red means this position is less likely to find this amino acid. For the ubiquitylated segments of the plant, it is more likely that arginine (R) will be found around the ubiquitylated lysine, especially on the 1st to 8th and −9th to −5th positions. In addition, it is clear that histidine (H), cysteine (C), and tryptophan (W) hardly appeared around the ubiquitylated lysine. The feature patterns in fungi and animals are different. For fungi, there is also some lysine (K) often appearing in the preorder of the ubiquitylated lysine, especially on the −9th to −1st position with M more than 0.75. However, lysine (K), which is followed by another lysine (K) in the next position usually is not ubiquitylated with M less than −0.75. For animals, we can find that the glutamic acid (E) more likely appeared on the −1st and −2nd positions near the ubiquitylated lysine with M of more than 0.75, but it less likely to appear on 1st and 2nd positions. The features of specific amino acid distribution in each position also differ in different species.

#### Two Sample Logo

**FIGURE 7:**
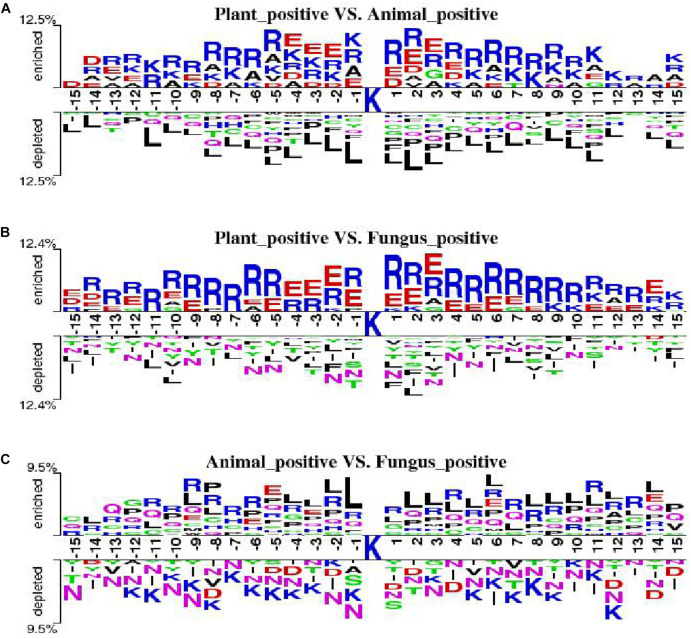
Comparison of the Two Sample Logo of three species. **(A)** Two Sample Logo of positive samples between plants and animals. **(B)** Two Sample Logo of positive samples between plants and fungi. **(C)** Two Sample Logo of positive samples between animals and fungi.

We employ the Two Sample Logo to show the differences of amino acid distribution in each position between ubiquitylated fragments from different species. The larger fonts denote the amino acid that is more likely to appear in this position with statistical significance. As shown in Figure 7A, we set the plant ubiquitylated fragments as positive samples and the animal ubiquitylated fragments as negative samples. We can see that more arginine (R), glutamic acid (E), aspartic acid (D), and alanine (A) appeared around the ubiquitylated lysine in the plants than in the animals, while it is less likely to find leucine (L). Then we set the plant ubiquitylated fragments as positive samples and the animal ubiquitylated fragments as negative samples, which are shown in Figure 7B. It is obvious that arginine (R) and glutamic acid (E) are more likely to appear around the ubiquitylated lysine in plants. As for the comparison of ubiquitylated fragments between animals and fungi, there were no obvious patterns except that there is more leucine (L) around the ubiquitylated lysine (Figure 7C).

According to the analyses above, the sequence features of the ubiquitylated fragments are really different between these three species. It is significant to build a ubiquitylation site prediction model for a single species, which can avoid the interference of feature differences from other species.

### Model Performance Evaluation

The proposed word embedding and CNN-based ubiquitination prediction model is evaluated through a validation test scheme. A 10-fold cross-validation is carried out on the training set for the fine-tuning of the hyper-parameters, as well as for evaluating the reliability of the model. In order to make the experiment results statistically significant, five repeated runs were conducted for each fold cross validation; the mean and standard deviation of the 50 results were regarded as the final result. The independent testing set was used for generalization evaluation and performance comparison with the baseline method. The confusion matrix of the prediction model is shown in Table 1, and the performance evolution indexes are defined as follows:

**TABLE 1:** Confusion matrix of ubiquitylated site prediction model.

	Predicted positive (Ub)	Predictive negative (non-Ub)
Actual positive (Ub)	True positive (TP)	False negative (FN)

Actual negative (non-Ub)	False positive (FP)	True negative (TN)

(a) Accuracy that indicates the proportion of correctly classified subjects among the whole subset
  $$\text{Accuracy}=\frac{TP+TN}{TP+TN+FP+FN}$$
(b) Precision that quantifies the proportion of samples correctly classified among the classification
  $$\text{Precision}=\frac{TP}{TP+FP}$$
(c) Recall is the fraction of relevant instances that have been retrieved over the total amount of relevant instances
  $$\text{Recall}=\frac{TP}{TP+FN}$$
(d) F-score considers both the precision and recall and evaluate the model performance synthetically
  $$F-\text{Score}=\frac{2\times \text{Precision}\times \text{Recall}}{\text{Precision}+\text{Recall}}$$

We first compared the proposed model performance with different tuning options through the 10-fold cross-validation scheme. Mean and standard deviation results of the cross validation are calculated, and the comparison results are shown in Table 2. The best performance with a mean accuracy of 78.1% and an F-score of 0.782 is given by the proposed model, which combines the transfer word-embedding mechanism and multilayer CNN. By contrast, the traditional one-hot sequence encoding method combined with a 2D CNN classifier obtains the worst performance with only a mean accuracy of 62.3% and an F-score of 0.647. This is mainly because the one-hot encoding matrix is sparse, and the conventional filters cannot capture useful sequence features. The pretrained word2vec encoding model without supervised weights updating also received a poor performance with a mean accuracy of 68.5% and an F-score of 0.6771. The word2vec model was trained on original plant protein sequences, which learned the amino acid bigram patterns of plants. However, without the fine-tuning process, the fixed weights cannot be adjusted to fit the ubiquitination site prediction task well. In addition, the supervised embedding layer with randomly initialized parameters also got a general performance; the effort of pretraining is obvious in ubiquitination site prediction in our proposed method. What is more, our results suggest that the recurrent neural network (RNN) does not contribute much to ubiquitination site prediction; this may because the distant sequence correlation modeling is not useful for this task.

**TABLE 2:** Cross validation performance comparison between different deep structures and feature encondings.

Model tuning	Accuracy	Precision	Recall	F-score
One-hot encoding + 2D convolutional neural network (CNN)	0.623 ± 0.037	0.662 ± 0.028	0.636 ± 0.019	0.647 ± 0.021
Embedding layer + CNN	0.732 ± 0.006	0.745 ± 0.011	0.692 ± 0.024	0.716 ± 0.029
Fixed word2vec + CNN	0.685 ± 0.024	0.701 ± 0.019	0.653 ± 0.015	0.677 ± 0.022
Transfer embedding + recurrent neural network (RNN)	0.743 ± 0.012	0.749 ± 0.004	0.716 ± 0.017	0.729 ± 0.015
Proposed method	0.782 ± 0.008	0.791 ± 0.013	0.785 ± 0.011	0.782 ± 0.016

### Independent Testing Performance

**FIGURE 8:**
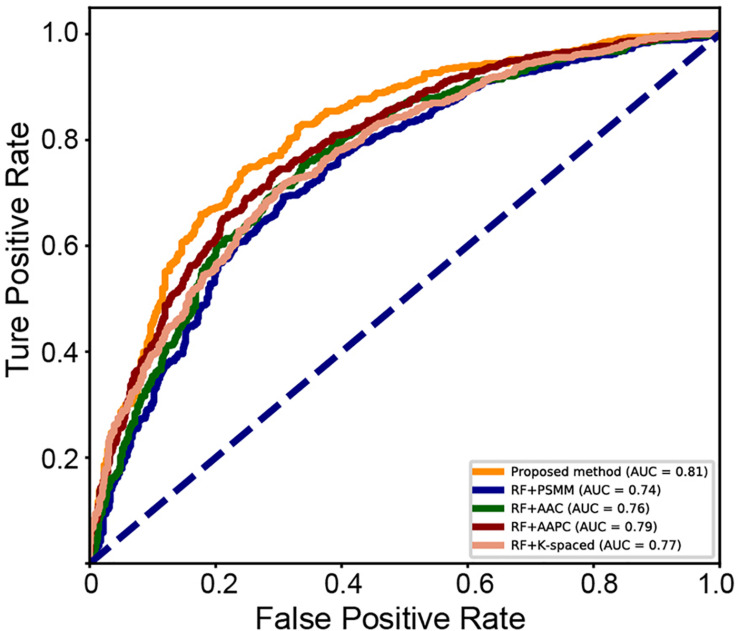
ROC curve of the different methods on the testing set.

A series of sequence features were extracted for modeling, including AAC, AAPC, the CKSAAPs, as well as the position-specific scoring matrix (PSSM). Our experiments indicated that the random forest (RF) model outperform other popular algorithms on all these predefined features. Table 3 shows the comparison between the proposed model and traditional feature-based random forest method on the testing set. The proposed model achieved the best performance with a mean accuracy of 75.6% and an F-score of 0.749. The random forest model also achieved an acceptable performance based on features of k-spaced amino acid pairs, with a mean accuracy of 73.6% and an F-score of 0.717. The PSSM represented the evolutionary profile of the protein sequence; the RF based on the PSSM features can achieved a mean accuracy of 71.1% and an F-score of 0.6942. Then as shown in Figure 8, we plotted the ROC curve with AUC of these RF-based model and our model. The proposed model is obvious, overall, in terms of the ROC curve with an 0.81 AUC, which indicates that the developed classifier has high confidence on plant ubiquitination site prediction.

**TABLE 3:** Performance comparison between different methods on the testing set.

Method	Accuracy	Precision	Recall	F-score
Random forest (RF) with amino acid composition (AAC)	0.703 ± 0.012	0.685 ± 0.026	0.703 ± 0.019	0.694 ± 0.022
RF with amino acid pairwise composition (AAPC)	0.711 ± 0.008	0.706 ± 0.017	0.679 ± 0.021	0.692 ± 0.031
RF with *k*-spaced AAP (*k* = 5)	0.736 ± 0.006	0.721 ± 0.009	0.714 ± 0.015	0.717 ± 0.019
RF with position-specific scoring matrices (PSMM)	0.722 ± 0.014	0.718 ± 0.008	0.706 ± 0.025	0.713 ± 0.018
Proposed method	0.756 ± 0.006	0.733 ± 0.015	0.767 ± 0.017	0.749 ± 0.009

In order to evaluate the generalization of the proposed model, we also collected data from the dbPTM and iPTMnet databases as an extra testing set. The dbPTM (Huang et al., 2019) and iPTMnet (Huang et al., 2018) contain 107 and 50 proteins of *A. thaliana*, respectively. The CD-HIT, with 30% identity, was employed to remove the redundant protein fragments and eliminate homology bias with the PLMD training data. Finally, 91 positive and 217 negative fragments were used for extra testing. The optimal model in cross validation achieved an accuracy of 74.2%, precision of 73.1%, recall of 73.7%, and F-score of 0.733. The proposed model can also achieve equal performance on other datasets.

### Comparison With Other Prediction Tools

We compared the performance of the proposed method with other popular ubiquitylation prediction tools on the independent set. For UbPred (Xiang et al., 2013), iUbiq-Lys (Qiu et al., 2015), and Ubisite (Huang et al., 2016), we uploaded our testing data to their website and counted the confusion matrix of output results to compute the performance indexes. For the Deep ubiquitylation (He et al., 2018) and DeepUbi (Fu et al., 2019), we reproduced their proposed structure with Keras, as well as training steps through our data, then calculated the evaluation indexes. As shown in Table 4, our proposed method achieved a balanced and reasonable performance with a mean precision of 73.3%, recall of 76.7%, and F-score with 0.749, although it only achieved a mean accuracy of 75.6%. It can be found that the iUbiq-Lys and Ubisite yielded a high recall and a poor precision, which means that these tools are more likely to classify the suspected samples as positive. Compared with Deep ubiquitylation, the first deep learning-based tool, our method achieved a better overall performance, which is mainly because the word-embedding scheme is more effective to extract the sequence features. The proposed method also outperformed the DeepUbi to some extent because the transfer learning-based method can capture the sequence pattern of plant proteins with word2vec model and the weights of embedding layer just fine-tuned around the pretrained value. In addition, the DeepUbi did not achieve the performance they claimed; this is mainly because the testing experiments are curried out on our plant data with on a small scale. Their proposed structure may need a larger training set to achieve optimal performance due to their training of the embedding layer from a random initial value. Overall, compared with popular tools and methods, our proposed model achieved a better performance on plant ubiquitylation site prediction.

**TABLE 4:** Performance comparison with other prediction tools.

Tool	Accuracy	Precision	Recall	F-score
UbPred (Xiang et al., 2013)	0.719	0.626	0.738	0.678
iUbiq-Lys (Qiu et al., 2015)	0.799	0.563	0.837	0.671
Ubisite (Huang et al., 2016)	0.752	0.596	0.794	0.681
Deep Ub (He et al., 2018)	0.683 ± 0.021	0.674 ± 0.018	0.703 ± 0.011	0.687 ± 0.024
DeepUbi (Fu et al., 2019)	0.739 ± 0.014	0.733 ± 0.011	0.741 ± 0.021	0.734 ± 0.011
Proposed method	0.756 ± 0.006	0.733 ± 0.015	0.767 ± 0.017	0.749 ± 0.009

Then predictions were conducted on two types of single plant protein: one contains ubiquitylated substrate sites, and the other has no ubiquitylated sites. The proposed model was compared with three popular ubiquitylation prediction tools, which provide websites for sequence input. The ubiquitylated protein was selected from an independent testing set randomly, and the protein that does not contain a ubiquitylated substrate site was selected from Uniport with no ubiquitylation sites reported. As shown in Table 5, the protein with Uniport AC O23063 contains 47 lysine and the positions of 142, 222, 225 are ubiquitylated (Walton et al., 2016). The iUbiq-Lys predicted five ubiquitylated sites, and only one is correct. The UbPred predicted one ubiquitylated site with other two false positive results. The Ubisite identified two sites successfully, while the proposed model can predict all the ubiquitylated sites correctly. It should be noted that the 363 position predicted by the proposed model is a false-positive sample; the performance of the proposed model still has room for improvement for some fragments. The protein with Uniport AC O03042 contains 24 lysine but no ubiquitylated site among them. The UbPred and Ubisite provided wrong predictions, while the iUbiq-Lys and the proposed model can classify them as non-ubiquitylated sites.

**TABLE 5:** Performance comparison with other tools on two types of single protein.

UniProt AC	Organism	Sequence length	Number of lysine	Reported ubiquitylated sites	Predicted ubiquitylated sites	
					Tools	Results
O23063	*Arabidopsis thaliana* (Mouse-ear cress)	364	47	142; 222; 225	iUbiq-Lys	3; 4; 103; 217; 225; 363
					UbPred	98; 142; 197
					Ubisite	142; 225; 265; 297
					Proposed model	142; 222; 225; 363
O03042	*Arabidopsis thaliana* (Mouse-ear cress)	479	24	None	iUbiq-Lys	None
					UbPred	8; 32; 201; 356; 474
					Ubisite	474
					Proposed model	None

The proposed model outperforms traditional machine learning and deep structure mainly because of its two novel characteristics. First, contrastive analyses found pattern differences of ubiquitylated fragments between the three species. Modeling for proteins from a single species can avoid the interference of feature differences from other species. Second, the transfer learning mechanism was employed to pretrain the embedding layer through the original plant protein sequence by the word2vec method, which can capture the sequence features of plant proteins and vectorize them. The Euclidean distance of vectors can reflect the relative position information of the amino acids. The embedding layer can capture the spatial features of amino acids in the pretraining process. So, the model is appropriate for the plant ubiquitination site prediction and achieved a better performance.

## Conclusion

In this work, we analyzed the sequence features of ubiquitylated protein from plants, animals, and fungi, respectively, then indicated the feature pattern differences between these features. We found that the amino acid distribution around the ubiquitylated lysine of plants differ from other species obviously, such as the clustering of arginine (*R*). The species of the plant was selected as the research target for modeling. A novel transfer learning-based word-embedding model training scheme was proposed. The original plant protein sequence was used for pretraining of the word2vec network through the skip-gram model, then the optimized parameter transfer as the initial weights of the embedding layer, fine-tuning with the subsequent layers together. The multilayer CNN was employed as a classifier and achieved acceptable performance for plant ubiquitination site prediction. Compared with related prediction tools, our method performs excellent suitability for plant ubiquitination site prediction. Considering the pattern differences between different species, in future work, we will integrate more data and establish species-specialized tools for ubiquitination site prediction.

## Data Availability Statement

All datasets presented in this study are included in the article/supplementary material.

## Author Contributions

T-YL conceived and headed this project. HW acquired the data, conducted the modeling work, and performed the experiments. ZW analyzed features and helped in the model evaluation works. ZL helped in the data collection and curation. All authors participated in writing or revising the manuscript.

## Conflict of Interest

The authors declare that the research was conducted in the absence of any commercial or financial relationships that could be construed as a potential conflict of interest.

## References

[B1] Cai Y. Huang T. Hu L. Shi X. Xie L. Li Y. (2012). Prediction of lysine ubiquitination with mRMR feature selection and analysis. *Amino Acids* 42 1387–1395. 10.1007/s00726-011-0835-0 21267749

[B2] Chen Z. Zhou Y. Song J. Zhang Z. (2013). hCKSAAP_UbSite: improved prediction of human ubiquitination sites by exploiting amino acid pattern and properties. *Biochim. Biophys. Acta* 1834 1461–1467. 10.1016/j.bbapap.2013.04.006 23603789

[B3] Du L. Wang Y. Song G. Lu Z. Wang J. (2018). “Dynamic network embedding: an extended approach for skip-gram based network embedding,” in *Proceedings of the Twenty-Seventh International Joint Conference on Artificial Intelligence*, New York, NY: IJCAI, 2086–2092.

[B4] Fu H. Yang Y. Wang X. Wang H. Xu Y. (2019). DeepUbi: a deep learning framework for prediction of ubiquitination sites in proteins. *BMC Bioinformatics* 20:86. 10.1186/s12859-019-2677-9 30777029 PMC6379983

[B5] Hamid M.-N. Friedberg I. (2018). Identifying antimicrobial peptides using word embedding with deep recurrent neural networks. *Bioinformatics* 35 2009–2016. 10.1093/bioinformatics/bty937 30418485 PMC6581433

[B6] He F. Wang R. Li J. Bao L. Zhao X. (2018). Large-scale prediction of protein ubiquitination sites using a multimodal deep architecture. *BMC Syst. Biol.* 12(Suppl. 6):109. 10.1186/s12918-018-0628-0 30463553 PMC6249717

[B7] Hoeller D. Hecker C.-M. Dikic I. (2006). Ubiquitin and ubiquitin-like proteins in cancer pathogenesis. *Na. Rev. Cancer* 6 776–788. 10.1038/nrc1994 16990855

[B8] Hua L. Quan C. (2016). A shortest dependency path based convolutional neural network for protein-protein relation extraction. *Biomed. Res. Int.* 2016:8479587. 10.1155/2016/8479587 PMC4963603 27493967

[B9] Huang C. H. Su M. G. Kao H. J. Jhong J. H. Weng S. L. Lee T. Y. (2016). UbiSite: incorporating two-layered machine learning method with substrate motifs to predict ubiquitin-conjugation site on lysines. *BMC Syst. Biol.* 10(Suppl. 1):6. 10.1186/s12918-015-0246-z 26818456 PMC4895383

[B10] Huang H. Arighi C. N. Ross K. E. Ren J. Li G. Chen S.-C. (2018). iPTMnet: an integrated resource for protein post-translational modification network discovery. *Nucleic Acids Res.* 46 D542–D550. 29145615 10.1093/nar/gkx1104 PMC5753337

[B11] Huang K.-Y. Lee T.-Y. Kao H.-J. Ma C.-T. Lee C.-C. Lin T.-H. (2019). dbPTM in 2019: exploring disease association and cross-talk of post-translational modifications. *Nucleic Acids Res.* 47 D298–D308. 30418626 10.1093/nar/gky1074 PMC6323979

[B12] Jyun-Rong W. Wen-Lin H. Ming-Ju T. Kai-Ti H. Hui-Ling H. Shinn-Ying H. (2016). ESA-UbiSite: accurate prediction of human ubiquitination sites by identifying a set of effective negatives. *Bioinformatics* 33 661–668. 10.1093/bioinformatics/btw701 28062441

[B13] Li W. Godzik A. (2006). Cd-hit: a fast program for clustering and comparing large sets of protein or nucleotide sequences. *Bioinformatics* 22 1658–1659. 10.1093/bioinformatics/btl158 16731699

[B14] Lu D. Lin W. Gao X. Wu S. Cheng C. Avila J. (2011). Direct ubiquitination of pattern recognition receptor FLS2 attenuates plant innate immunity. *Science* 332 1439–1442. 10.1126/science.1204903 21680842 PMC3243913

[B15] Marino D. Rivas P. S. (2012). Ubiquitination during plant immune signaling. *Plant Physiol.* 160 15–27. 10.1104/pp.112.199281 22689893 PMC3440193

[B16] Mikolov T. Chen K. Corrado G. Dean J. (2013). Efficient estimation of word representations in vector space. *arXiv.* [preprint]. Avaliable at: https://arxiv.org/abs/1301.3781 (accessed June 13, 2020).

[B17] Neishi M. Sakuma J. Tohda S. Ishiwatari S. Yoshinaga N. Toyoda M. (2017). “A bag of useful tricks for practical neural machine translation: embedding layer initialization and large batch size,” in *Proceedings of the 4th Workshop on Asian Translation (WAT2017)*), Taipei, 99–109.

[B18] Nguyen V. N. Huang K. Y. Huang C. H. Chang T. H. Bretaña N. Lai K. (2015). Characterization and identification of ubiquitin conjugation sites with E3 ligase recognition specificities. *BMC Bioinformatics* 16(Suppl.1):S1. 10.1186/1471-2105-16-S1-S1 25707307 PMC4331700

[B19] Nguyen V. N. Huang K. Y. Huang C. H. Lai K. R. Lee T. Y. (2016). A new scheme to characterize and identify protein ubiquitination sites. *IEEE/ACM Trans. Comput. Biol. Bioinform.* 14 393–403. 10.1109/tcbb.2016.2520939 26887002

[B20] Popovic D. Vucic D. Dikic I. (2014). Ubiquitination in disease pathogenesis and treatment. *Nat. Med.* 20 1242–1253. 10.1038/nm.3739 25375928

[B21] Qiu W.-R. Xiao X. Lin W.-Z. Chou K.-C. (2015). iUbiq-Lys: prediction of lysine ubiquitination sites in proteins by extracting sequence evolution information via a gray system model. *J. Biomol. Struc. Dyn.* 33 1731–1742. 10.1080/07391102.2014.968875 25248923

[B22] Radivojac P. Vacic V. Haynes C. Cocklin R. R. Mohan A. Heyen J. W. (2010). Identification, analysis, and prediction of protein ubiquitination sites. *Proteins Struc. Funct. Bioinform.* 78 365–380. 10.1002/prot.22555 19722269 PMC3006176

[B23] Schmidhuber J. (2015). Deep learning in neural networks: an overview. *Neural Netw.* 61 85–117. 10.1016/j.neunet.2014.09.003 25462637

[B24] Tu Y. Chen C. Pan J. Xu J. Wang C. Y. (2012). The ubiquitin proteasome pathway (UPP) in the regulation of cell cycle control and DNA damage repair and its implication in tumorigenesis. *Int. J. Clin. Exp. Pathol.* 5 726–738. 23071855 PMC3466981

[B25] Tung C.-W. Ho S.-Y. (2008). Computational identification of ubiquitylation sites from protein sequences. *BMC Bioinformatics* 9:310. 10.1186/1471-2105-9-310 18625080 PMC2488362

[B26] Vacic V. Iakoucheva L. M. Radivojac P. (2006). Two sample logo: a graphical representation of the differences between two sets of sequence alignments. *Bioinformatics* 22 1536–1537. 10.1093/bioinformatics/btl151 16632492

[B27] Walton A. Stes E. Cybulski N. Van Bel M. Iñigo S. Durand A. N. (2016). It’s time for Some “Site”-Seeing: novel tools to monitor the ubiquitin landscape in arabidopsis thaliana. *Plant Cell* 28 6–16. 10.1105/tpc.15.00878 26744219 PMC4746685

[B28] Weissman A. M. (2001). Themes and variations on ubiquitylation. *Nat. Rev. Mol. Cell Biol.* 2 169–178. 10.1038/35056563 11265246

[B29] Xiang C. Qiu J. D. Shi S. P. Suo S. B. Huang S. Y. Liang R. P. (2013). Incorporating key position and amino acid residue features to identify general and species-specific Ubiquitin conjugation sites. *Bioinformatics* 13 1614–1622. 10.1093/bioinformatics/btt196 23626001

[B30] Xu H. Zhou J. Lin S. Deng W. Zhang Y. Xue Y. (2017). PLMD: An updated data resource of protein lysine modifications. *J. Genet. Genomics* 44 243–250. 10.1016/j.jgg.2017.03.007 28529077

[B31] Yamada T. Murata D. Adachi Y. Itoh K. Kameoka S. Igarashi A. (2018). Mitochondrial stasis reveals p62-mediated ubiquitination in Parkin-independent mitophagy and mitigates nonalcoholic fatty liver disease. *Cell Metab.* 28 588.e5–604.e5. 30017357 10.1016/j.cmet.2018.06.014 PMC6170673

